# Regeneration of tracheal neotissue in partially decellularized scaffolds

**DOI:** 10.1038/s41536-023-00312-4

**Published:** 2023-07-12

**Authors:** Zheng Hong Tan, Sayali Dharmadhikari, Lumei Liu, Jane Yu, Kimberly M. Shontz, Jacob T. Stack, Christopher K. Breuer, Susan D. Reynolds, Tendy Chiang

**Affiliations:** 1 Center of Regenerative Medicine, Abigail Wexner Research Institute, Nationwide Children’s Hospital, Columbus, OH USA; 2 College of Medicine, The Ohio State University, Columbus, OH USA; 3 Center for Perinatal Research, Nationwide Children’s Hospital, Columbus, OH USA; 4 Department of Pediatric Surgery, Nationwide Children’s Hospital, Columbus, OH USA; 5 Department of Pediatric Otolaryngology, Nationwide Children’s Hospital, Columbus, OH USA

**Keywords:** Respiratory tract diseases, Preclinical research, Experimental models of disease, Paediatric research

## Abstract

Extensive tracheal injury or disease can be life-threatening but there is currently no standard of care. Regenerative medicine offers a potential solution to long-segment tracheal defects through the creation of scaffolds that support the generation of healthy neotissue. We developed decellularized tracheal grafts (PDTG) by removing the cells of the epithelium and lamina propria while preserving donor cartilage. We previously demonstrated that PDTG support regeneration of host-derived neotissue. Here, we use a combination of microsurgical, immunofluorescent, and transcriptomic approaches to compare PDTG neotissue with the native airway and surgical controls. We report that PDTG neotissue is composed of native tracheal cell types and that the neoepithelium and microvasculature persisted for at least 6 months. Vascular perfusion of PDTG was established within 2 weeks and the graft recruited multipotential airway stem cells that exhibit normal proliferation and differentiation. Hence, PDTG neotissue recapitulates the structure and function of the host trachea and has the potential to regenerate.

## Introduction

The trachea is a large, cartilaginous organ that conducts air to the lung parenchyma. Diseases of the large airway can arise from or result in a loss of patency, mucociliary clearance, and immunologic function^1,2^. Further, tracheal stenosis can cause life-threatening airflow restriction and is the most common indication for reconstructive surgery^3^.

Current airway reconstruction methods rely on endoscopic techniques, the manipulation of existing tracheal tissue by way of expansion, and resection techniques. Unfortunately, no standard of care exists for large segment defects or tracheal agenesis. Regenerative medicine has the potential to create a graft that grows with the patient. To this end, tracheal tissue engineering has explored the use of synthetic and biological grafts for airway replacement^4^. Approaches to create tissue-derived grafts have used decellularization, a method that removes donor cells and preserves a non-immunogenic, 3-dimensional scaffold of extracellular matrix^4–7^. Although complete decellularization compromises the graft’s mechanical properties, partial decellularization approaches can overcome this issue by preserving tracheal cartilage and chondrocytes^8–11^.

We created a partially decellularized tracheal graft (PDTG) and reported that it supported the generation of host-derived neotissue in vivo^11–13^. Within 1 month, the PDTG was lined by an epithelium, microvasculature, and was patent. In this study, we used a combination of microsurgical, immunofluorescent, and transcriptomic approaches to quantify the cell types within the tracheal neotissue at later time points and explored progenitor-progeny relationships within the epithelium.

## Results

### Partial decellularization preserves tracheal cartilage, extracellular matrix, and structure

**Fig. 1:**
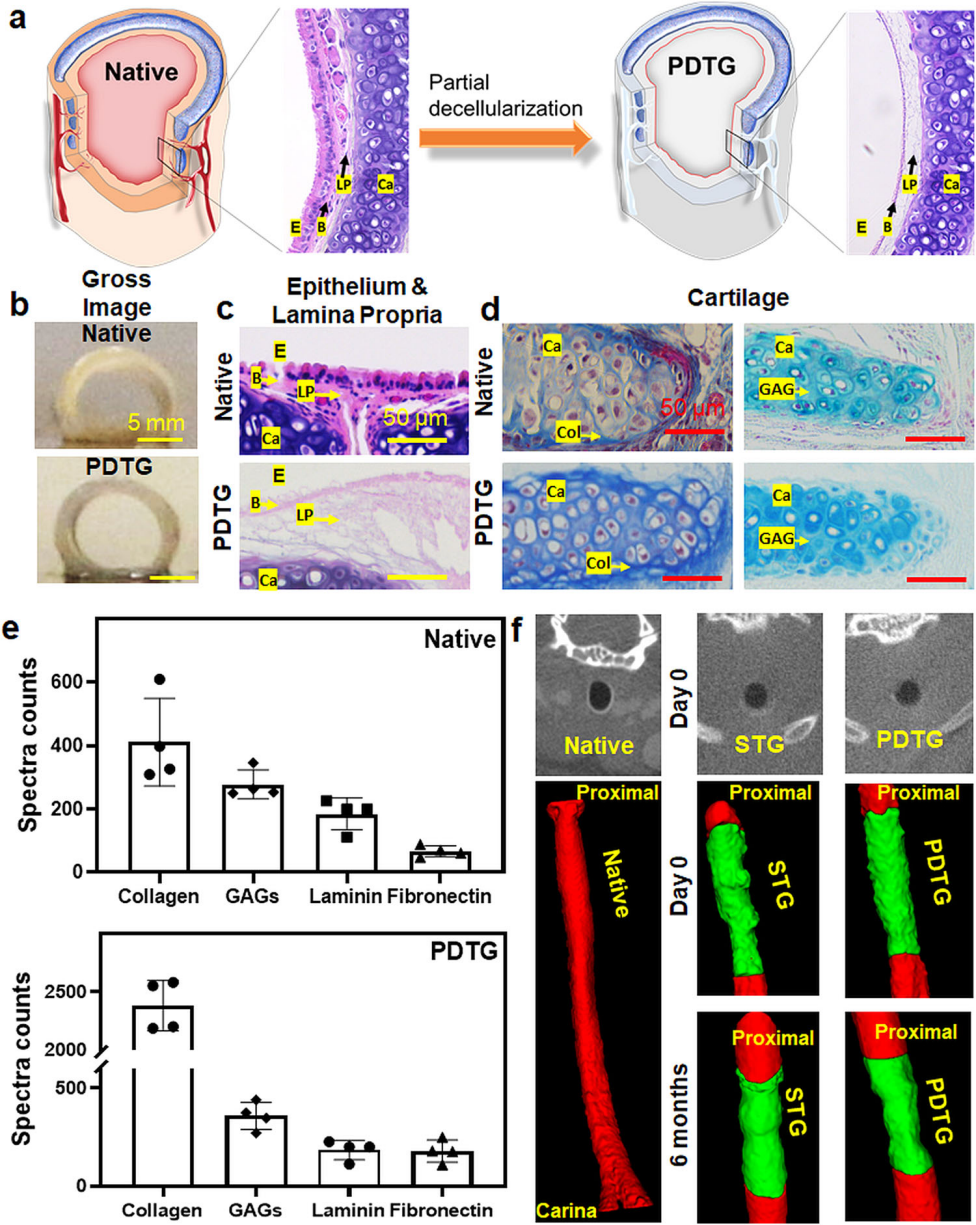
Partially decellularized tracheal graft characterization. **a** Graphical representation of native and partially decellularized trachea. Representative images (*n* = 4) of native and partially decellularized tracheas: (**b**) gross, (**c**) Partial decellularization removes all cells from the luminal surface and lamina propria. H&E. **d** Partial decellularization preserves cartilage extracellular matrix. Masson’s trichrome staining of the cartilage and Alcian blue staining of the cartilage region. **e** Mass spectrometric analysis of ECM proteins in the native and partially decellularized trachea. **f** Representative microCT 3D reconstructions (*n* = 4) of native, syngeneic, and partially decellularized trachea at day 0 and 6 months post tracheal replacement. (E Epithelium, B Basement membrane, LP Lamina Propria, Ca Cartilage, Col Collagen, GAG Glycosaminoglycans).

Partial decellularization was performed on 5-ring tracheal segments (rings 3–8) that were recovered from C57Bl/6 J mice (Fig. 1a). Gross assessment revealed that this process preserved the C-shaped cartilage rings and chondrocytes while removing all cells from the epithelium and lamina propria (Fig. 1a–d). The extracellular matrix (ECM) of the tracheal basement membrane and lamina propria was preserved (Fig. 1c). Assessment of the cartilage using Masson’s Trichrome and Alcian Blue staining revealed persistence of collagen and glycosaminoglycans respectively (Fig. 1d).

Mass spectrometry was used to catalogue the proteins present in PDTG and native trachea. The native trachea contained 5073 proteins while PDTG contained 1893 proteins. These data showed that 62.6% of proteins were removed by the decellularization process and gene ontology analysis indicated that most of these proteins were cell associated. Key ECM proteins including collagen, glycosaminoglycans, laminin and fibronectin were present in both PDTG and native trachea (Fig. 1e).

The intent behind the preservation of tracheal cartilage during partial decellularization is to retain graft mechanical properties. As a surrogate for graft mechanics, we performed orthotopic implantation and assessed graft patency after implantation with microCT, a method that is similar to the radiographic evaluation of children with tracheomalacia^14^ (Fig. 1f). PDTG was found to be patent at day 0 and 6 months after orthotopic implantation (Fig. 1f). None of the animals analyzed in this study exhibited signs of respiratory distress at the time of euthanasia.

### PDTG neotissue is composed of the same cell populations found in the native trachea

**Fig. 2:**
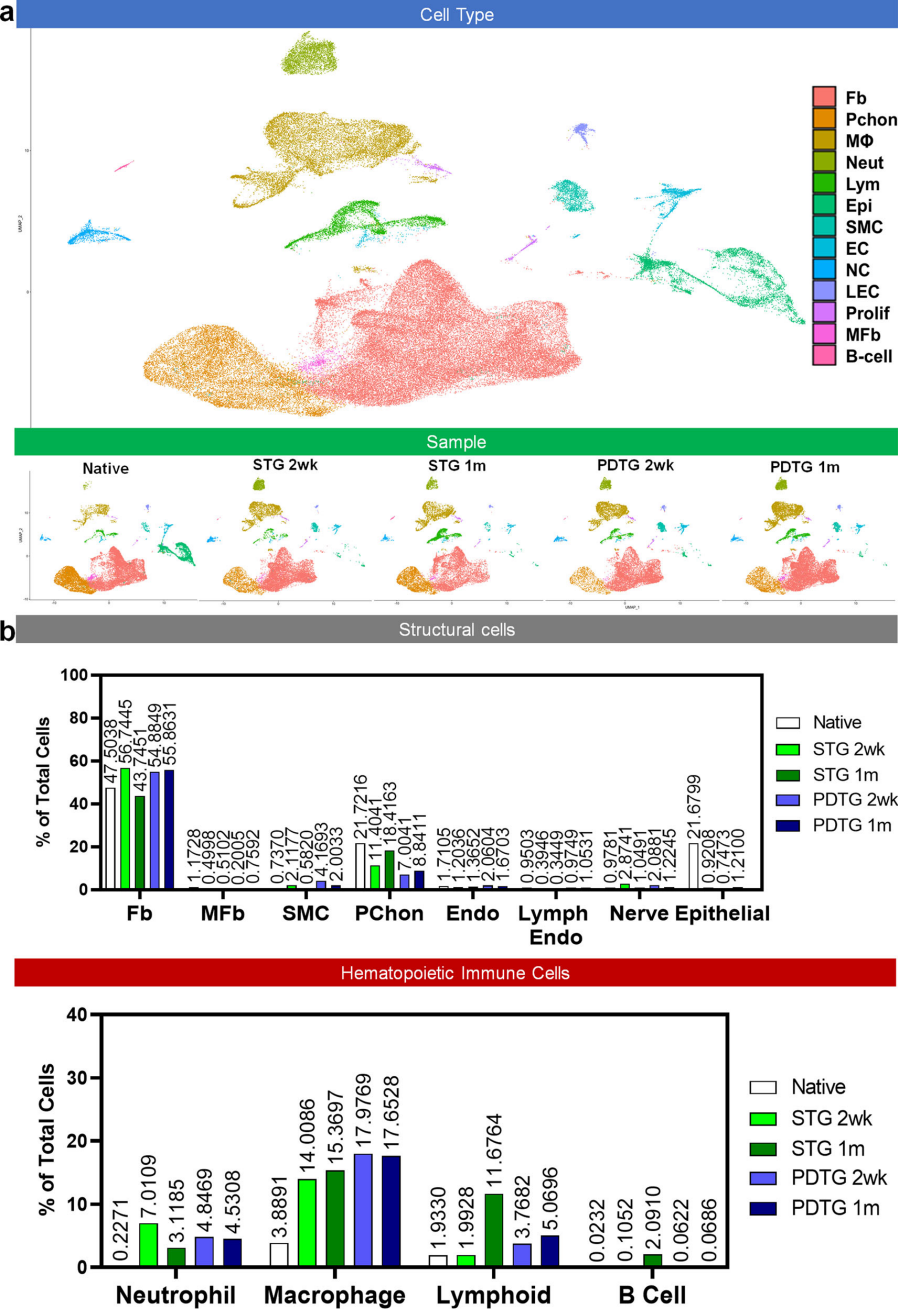
Single-cell RNA sequencing analysis. **a** Uniform Manifold Approximation and Projection (UMAP) visualization of the single cells in native, syngeneic tracheal transplants (STG) and partially decellularized tracheal grafts (PDTG) at 2 weeks and 1 month. MyoE Myoepithelial Cells, Fb Fibroblasts, PChon Perichondrial Cells, MΦ Macrophage, Neut Neutrophils, Lym Lymphoid Cells, Epi Epithelial Cells, SMC Smooth Muscle Cells, EC Endothelial Cells, NC Nerve Cells, LEC Lymph Endothelial Cells, Prolif Proliferating Cells. MFb Myofibroblasts, B-cell B Cells. **b** Proportion of Structural cell types and Hematopoietic immune cell types analyzed. The proportion of the particular cell type in a particular sample is labeled above the bars.

To comprehensively assess PDTG neotissue, we first examined native tracheal tissue and tracheal tissue after syngeneic tracheal replacement (STG) which served as surgical controls. Single cell suspensions from PDTG, STG, and native trachea were recovered via enzymatic digestion. This method did not digest cartilage. Transcriptomes were profiled using single-cell RNA sequencing methodologies. Cell identity was inferred using the expression of canonical marker genes (Supplemental Data 1). Automated cell type annotations drawn from independent databases agreed with manual cell classifications^15–17^. Enzymatic digestion of trachea was only effective at isolating cells from the epithelium and lamina propria. The most prevalent cell type found in the native trachea was the fibroblast (47.5%). Other abundant cell types included epithelial (21.7%), hematopoietic (6.07%), endothelial (1.71%), and smooth muscle (0.74%) (Fig. 2a).

We used the syngeneic tracheal transplant (STG) model to determine the impact of airway surgery on cellular diversity within the graft. Two weeks after implantation, transplanted syngeneic grafts contained all the cell types identified in the native trachea. The frequencies of fibroblasts (56.7%), endothelial (1.20%), and smooth muscle (2.11%) were similar to native trachea. However, epithelial cells were underrepresented (0.92%) and hematopoietic cells (23.1%) were overrepresented in transplanted syngeneic grafts. Macrophages were the most prevalent hematopoietic cell type. The relative frequencies of the various cell types did not vary between the 2- and 4-week time points.

PDTG neotissue was composed of the same cell types observed in the native and STG groups. In PDTG, the frequency of fibroblasts (54.9%), endothelial (2.06%), and smooth muscle cells (4.17%) was more similar to syngeneic tissue than native trachea. These differences extended to epithelial (1.21% PDTG, 0.92% STG, 21.7% native trachea) and hematopoietic (26.7% PDTG, 23.1% STG, 6.07% native trachea) cell frequency. Similar to STG, macrophages were the most prevalent hematopoietic cell type found in PDTG. Among surgical groups (PDTG and syngeneic transplant) the frequency of cell types did not vary between the 2-weeks and 1-month time points (Fig. 2b). Collectively, these data demonstrate that PDTG supports the reestablishment of native tracheal cell populations and that variations in cell type frequency were primarily due to the transplantation procedure rather than graft type.

### Host-derived tracheal neotissue contains a functional vasculature

**Fig. 3:**
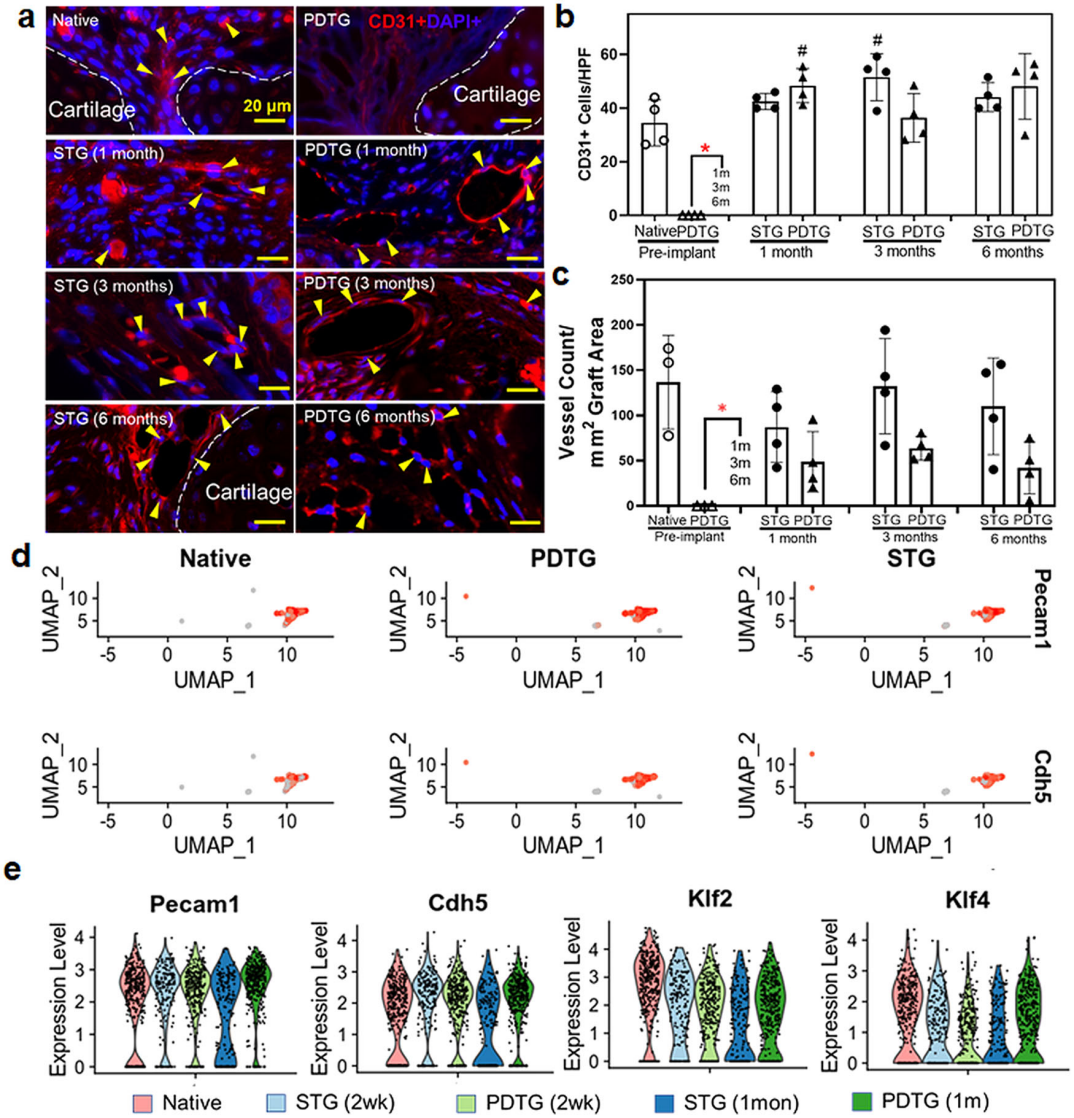
Vascularization in partially decellularized tracheal grafts. **a** Representative images of endothelial cells (CD31+, red) in native trachea, PDTG, and on STG and PDTG 1, 3, and 6 months post-implantation. Yellow arrows denote endothelial cells forming vessels in the trachea submucosa. White dotted lines denote adjacent tracheal cartilage (**b**) Quantification of CD31+ endothelial cells on the graft (cells/high powered field (HPF)). Native: 34.5 ± 8.59, PDTG (pre-implant): 0 ± 0, STG(1 m): 42.5.5 ± 3.00 PDTG(1 m): 48.4 ± 6.35, STG(3 m): 51.6 ± 8.74, PDTG(3 m): 36.4 ± 9.08, STG(6 m): 48.1 ± 12.2, PDTG(6 m): 44.1 ± 5.38. *n* = 4 for each group. Points indicate values for different trachea or PDTG. **c** Quantification of blood vessel count (blood vessels/mm^2^ graft area). # represents the difference (*p* < 0.05) compared to the native trachea. * represents the difference (*p* < 0.05) between pre-implant PDTG versus STG and PDTG groups. Native: 136 ± 51.8, PDTG (pre-implant): 0 ± 0, STG(1 m): 87.3 ± 39.0 PDTG(1 m): 48.8 ± 33.2, STG(3 m): 132 ± 52.6, PDTG(3 m): 63.7 ± 13.2, STG(6 m): 110 ± 53.5, PDTG(6 m): 42.0 ± 28.8. *n* = 4 for each group. Points indicate values for different trachea or PDTG. **d** Feature plots highlighting the canonical cell markers used to identify endothelial cell cluster identities. **e** Violin Plots demonstrating the expression of canonical endothelial cell markers (Pecam1, Cdh5) and endothelial flow markers (Klf2, Klf4). Data are presented as mean ± SD. ^#^*p* < 0.05 *p < 0.05 by unpaired Student’s t-test, one- or two-way ANOVA followed by Turkey’s post hoc test.

Endothelial cells (CD31+) are detected in tracheal neotissue as early as 2 weeks in vivo, forming tubular vessels that were filled with red blood cells (Fig. 3). Endothelial cell and vessel count of tracheal neotissue were comparable to syngeneic transplants (STG) throughout the study time points (Fig. 3a–c). Next, we used differential gene expression of endothelial cells to confirm vessel function. We found that endothelial cells in PDTG neotissue express Klf2 and Klf4, genes that are activated when subjected to flow shear stresses^18,19^ (Fig. 3d, e). These data indicate that functional vasculature with flow was established in PDTG by 2 weeks post implantation.

### PDTG regeneration restores the mucociliated airway epithelium

**Fig. 4:**
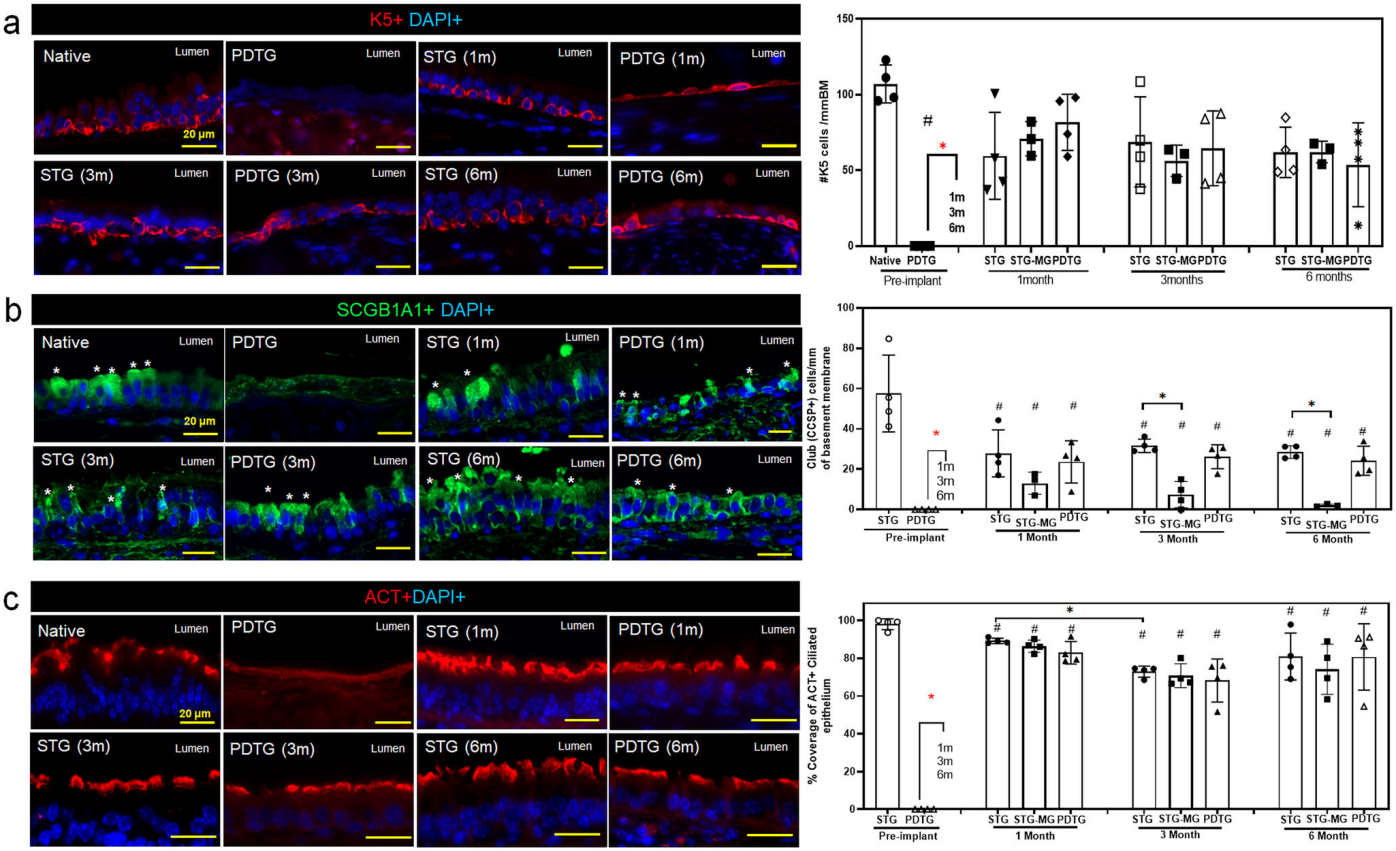
Epithelialization of partially decellularized tracheal grafts. **a** Representative images of basal cells (K5 + ) on STG and PDTG and quantification of K5+ basal cell density (cells/mm) on Native, STG, Midgraft region of STG (STG-MG), PDTG at 1-,3- and 6-month. Native: 107 ± 12.5, PDTG (pre-implant): 0 ± 0, STG(1 m): 59.5 ± 28.8, STG(1 m)-MG: 70.76 ± 11.26, PDTG(1 m): 81.6 ± 18.6, STG(3 m): 68.8 ± 29.7, STG(3 m)-MG: 56.28 ± 10.34, PDTG(3 m): 64.4 ± 24.7, STG(6 m): 61.8 ± 16.6, STG(6 m)-MG: 62.04 ± 7.089, PDTG(6 m): 53.6 ± 27.6. *n* = 4 for each group. Points indicate values for different trachea or PDTG. **b** Representative images of club (SCGB1A1+) cells on STG and PDTG (* denotes club cells) and quantification of SCGB1A1+ club cell density (cells/mm). Native: 57.5 ± 19.0, PDTG (pre-implant): 0 ± 0, STG(1 m): 27.8 ± 11.7, STG(1 m)-MG: 13.01 ± 5.488, PDTG(1 m): 23.6 ± 10.5, STG(3 m): 31.6 ± 3.30, STG(3 m)-MG: 7.341 ± 6.49, PDTG(3 m): 26.2 ± 5.92, STG(6 m): 28.5 ± 3.12, STG(6 m)-MG: 2.030 ± 0.683, PDTG(6 m): 24.21 ± 7.23. *n* = 4 for each group. Points indicate values for different trachea or PDTG. **c** Representative images of ciliated (ACT + ) cells on STG and PDTG and quantification of ACT+ ciliated cell coverage on graft (average % coverage of ACT). # - difference (*p* < 0.05) compared to the native trachea. * - difference (*p* < 0.05) between STG and PDTG groups. * - difference (*p* < 0.05) between pre-implant PDTG versus STG and PDTG groups. Native: 98.0 ± 2.92%, PDTG (pre-implant): 0 ± 0, STG(1 m): 89.2 ± 1.40%, STG(1 m)-MG: 86.33 ± 3.23%, PDTG(1 m): 82.9 ± 5.92%, STG(3 m): 72.8 ± 2.90%, STG(3 m)-MG: 70.79 ± 6.304%, PDTG(3 m): 68.2 ± 11.4%, STG(6 m): 80.9 ± 12.4%, STG(6 m)-MG: 74.19 ± 13.27%, PDTG(6 m): 80.7 ± 17.6% *n* = 4 for each group. Points indicate values for different trachea or PDTG. Data are presented as mean ± SD. ^#^*p* < 0.05 *p < 0.05 by unpaired Student’s t-test, one- or two-way ANOVA followed by Turkey’s post hoc test.

We previously reported that PDTG neotissue is composed of a differentiated epithelium comprised of multiciliated cells and basal cells on day 28 post-implantation^11^. To evaluate epithelial differentiation at later time points, 3 and 6 months, PDTG were stained for basal (KRT5), club (SCGB1A1), and ciliated (Acetylated tubulin, ACT) cell markers (Fig. 4a–c). The frequency of basal cells did not differ among the three groups (Fig. 4a). In contrast, the frequencies of multiciliated and club cells in PDTG and syngeneic transplants were lower than that found in the native epithelium (Fig. 4b, c). Notably, a reduction in multiciliated and club cell frequency was detected in the mid-graft of STG, a region that is free from surgical manipulation. Moreover, multiciliated and club cell frequency did not differ between PDTG and syngeneic transplants (Fig. 4b, c). These data suggested that the main determinant of club and ciliated cell frequency was due to the reconstructive procedure rather than the graft type.

### Tracheal replacement induces basal cell activation

**Fig. 5:**
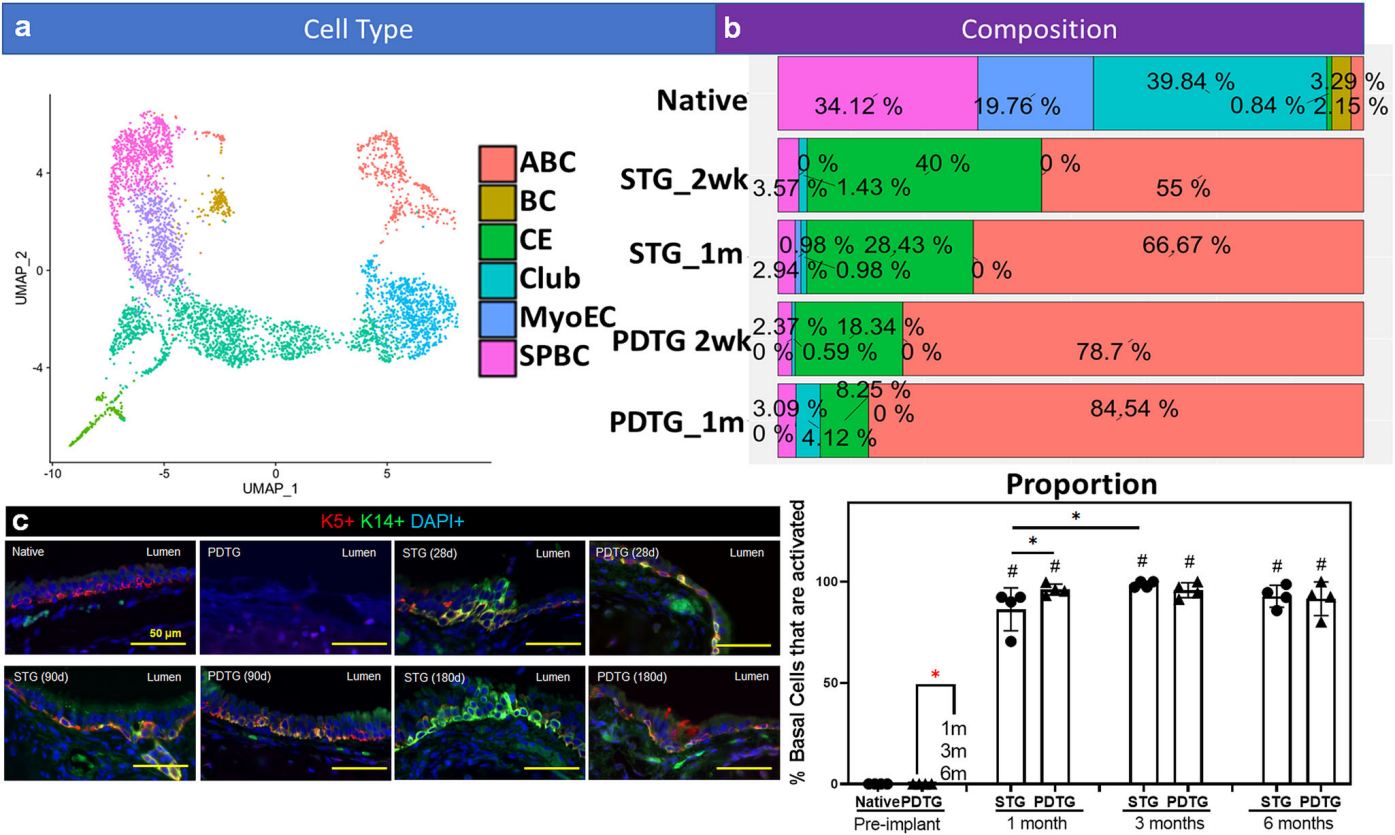
Basal cell activation in tracheal grafts. **a** UMAP demonstrating all epithelial cell types and (**b**) epithelial cell composition in native, syngeneic tracheal graft transplants (STG) and partially decellularized tracheal grafts (PDTG) at 2 weeks and 1 month. ABC Activated Basal Cell, BC Basal Cell, CE Ciliated Cell, Club Club Cell, MyoEC Myoepithelial Cell, SPCB Secretory Primed Basal Cell. **c** Representative images of activated basal cells (K5+/K14+) on STG and PDTG with quantification. Native: 0 ± 0, PDTG (pre-implant): 0 ± 0, STG(1 m): 86.3 ± 10.6%, PDTG(1 m): 96.1 ± 2.71%, STG(3 m): 98.6 ± 1.54%, PDTG(3 m): 95.8 ± 3.69%, STG(6 m): 92.8 ± 5.45%, PDTG(6 m): 91.5 ± 8.3%. *n* = 4 for each group. Points indicate values for different trachea or PDTG. Data are presented as mean ± SD. ^#^*p* < 0.05 *p < 0.05 by unpaired Student’s t-test, one- or two-way ANOVA followed by Turkey’s post hoc test.

Single cell RNA sequencing demonstrated that 2.15% of native tissue basal cells expressed an activated (KRT14+) phenotype. Syngeneic and PDTG neotissue contained a greater proportion of activated basal cells compared to native control and that all basal cells in these grafts were activated (Fig. 5a, b). To determine if basal cell activation persisted, the frequency of KRT14+ cells was determined at 3 and 6 months. (Fig. 5c) Quantification demonstrated that KRT14+ basal cells were significantly more frequent on STG and PDTG relative to native control. Although KRT14+ cell frequency was greater on PDTG than STG at the 1-month time point, differences were not detected at 3 or 6 months.

### Activated basal cells proliferate and are multipotential progenitors

**Fig. 6:**
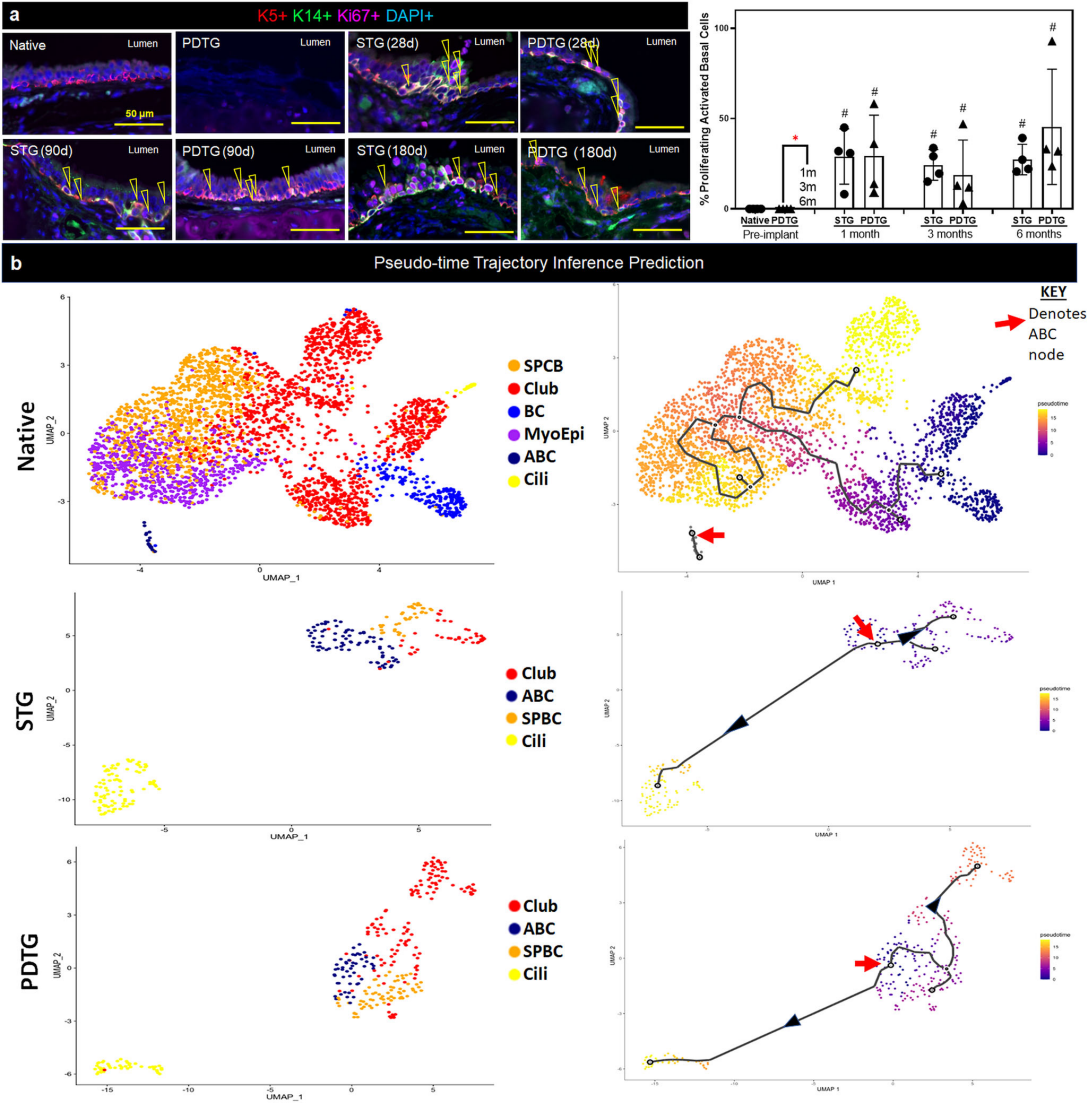
Self-renewing and multipotent basal cells in partially decellularized trachea grafts. **a** Images of proliferating activated basal progenitor cells (K5+/K14+/Ki67+) on STG and PDTG (∆) and quantification. Native: 0 ± 0, PDTG (pre-implant): 0 ± 0, STG(1 m): 29.0 ± 15.3%, PDTG(1 m): 29.4 ± 22.5%, STG(3 m): 24.4 ± 8.53%, PDTG(3 m): 18.8 ± 19.4%, STG(6 m): 27.4 ± 8.44%, PDTG(6 m): 45.4 ± 31.9% *n* = 4 for each group. Points indicate values for different trachea or PDTG. **b** Trajectory-inference analysis to determine cell-fate in native, STG and PDTG. SPCB Secretory Primed Basal Cell, Club Club Cell, BC= Basal Cell, MyoE Myoepithelial Cell, ABC Activated Basal Cell, Cili Ciliated Cell. Data are presented as mean ± SD. ^#^*p* < 0.05 *p < 0.05 by unpaired Student’s t-test, one- or two-way ANOVA followed by Turkey’s post hoc test.

To determine if the activated basal cells were proliferating, tissue sections were co-stained for KRT14 and Ki67. The frequency of mitotic-activated basal cells was significantly greater in both surgical groups but did not differ between the syngeneic transplant and PDTG (Fig. 6a). These data indicated that there was an ongoing regenerative process and that this was determined by the reconstructive procedure rather than the graft type.

We characterized progenitor-progeny relationships using single-cell RNA sequencing to determine if basal cell activation resulted in production of nascent ciliated and club cells. Trajectory inference analysis of native tracheal epithelial cells revealed that the basal cells are differentiating into club cell types. This is consistent with current understanding of basal cell turnover during homeostasis^20^. Activated basal cell populations in native trachea did not produce differentiated cell types. However, these activated basal cells are infrequent within the native trachea and single cell RNA sequencing might not be able to detect the progenitor-progeny relationships for these rare populations^21^. In contrast, the activated basal cells in both STG and PDTG generated ciliated and club cells (Fig. 6b). These data suggested that activated basal cells function as multipotential progenitors during tracheal replacement and indicated that their differentiation potential was similar to that described for basal cells that are involved in epithelial repair^22–24^.

## Discussion

As a solution for life-threatening tracheal injury and disease, we developed tracheal replacement using a partial decellularization techniques. This study extends our prior work by (1) using single cell RNAseq to measure the similarities and differences between native trachea and tracheal neotissue (2) using histological methods to measure the endurance of tracheal cell types in neotissue (3) define the cell fate of airway basal cells in epithelial regeneration and (4) determine if similarities from the native airway are attributed to surgical intervention or are graft dependent.

Single cell RNAseq analysis demonstrated increased immune cell infiltration in grafts following both syngeneic tracheal transplant and PDTG implantation. Both grafts exhibited a similar macrophage-dominant inflammatory response. With no histologic evidence of rejection and the congruence with syngeneic grafts, it is likely that the macrophage-dominant inflammatory response is due to the surgical procedure^25–28^. This conclusion was supported by our scRNAseq data which identified ongoing production of basal cell derived club and ciliated cells. Previous work suggests that this repair response may persist up to a year after surgery^29^.

Tracheal neotissue recapitulates the diversity of the native trachea by 2 weeks despite having an acellular epithelium and lamina propria at the time of implantation. This spatially appropriate recellularization of PDTG suggests the restoration of tracheal tissue, as evident from our analysis of tracheal vascularization and the repopulation of stromal cells. Further, the secretory ciliated epithelium is limited to the luminal surface of the graft, demonstrating spatially appropriate recellularization and implying mucosecretory clearance.

Creation of a functional epithelium with the capacity for proliferation and differentiation is crucial for curative tracheal replacement. The basal cell is the tissue-specific stem cell that maintains the tracheobronchial airway epithelium^30–33^. Following epithelial injury, basal cells are activated and serve as the source of the nascent club and ciliated cells which restore epithelial structure and function^22–24^. Previous work also indicated that human basal stem cells migrate in response to injury and that mouse basal stem cells recreate their niche in vitro^34–38^. We found that basal cells in tracheal neotissue make cell fate decisions that are similar to those detected in the syngeneic transplant and that these cells produce both club and ciliated cells. These data suggest the epithelialization mechanics of PDTG, mediated by stem cells that migrate onto the graft, exhibit the functions that are similar to basal cells in native tissue. This similarity demonstrates the potential application of PDTG for the next stages of pre-clinical study. The complementary approaches of histology and transcriptomic provided us with a comprehensive analysis of neotissue formation. We report that the PDTG scaffold recruits basal cells and supports differentiation of each cell type that is found in the native trachea. Further analysis of this process has the potential to identify the cues that are required for neotissue formation and therapeutic targets that play a role in airway healing. Additionally, a comprehensive understanding of how surgery regulates epithelial differentiation and function may lead to methods that improve graft function, particularly reestablishment of mucociliary clearance. Our findings and the literature indicate that the PDTG supports the formation of a neoepithelium with the ability to respond to future injury and grow with the pediatric patient.

Although our data indicate that tissue-specific stem cells inhabit the neoepithelium, stem cell mediated regeneration is typically completed by 2–4 weeks^22,30,31^. In contrast, the PDTG and STG grafts were lined by an epithelium that had less club and ciliated cells. Since both graft types harbored epithelial stem cells, protracted epithelial regeneration suggested that the surgical process altered the stem cell’s ability to produce differentiated cell types. Multilineage differentiation, as well as self-renewal, is regulated by the stem cell niche^37^. In the airway, the stem cell niche is comprised of tissue stem cell progeny, smooth muscle, fibroblasts, and/or endothelial cells^39–42^. Although we report that PDTG and STG were populated by similar connective and vascular cell types, it is possible that regeneration of tracheal tissues lacks appropriate temporal and/or spatial synchronization. Thus, surgical disruption of the epithelial mesenchymal trophic unit may result in maladaptation and long-term tissue repair^43^. This could be a shift in paradigm in the field of airway tissue engineering where much of the focus is on the scaffold or the host and the impact of surgery on bioengineered grafts is largely unexplored. Future research is needed to evaluate the interactions among tissue types in the regenerating graft and to assess alternative explanations for ciliated and club cell hypoplasia including cell death and loss of cellular identity^44^.

In conclusion, we established that PDTG supported spatially appropriate neotissue formation in vivo. PDTG perfusion was established by 2 weeks and the airway epithelium was populated by multipotential basal stem cells which generated a secretory-ciliated epithelium. Our findings support the use of partial decellularization to create airway grafts for long-segment airway reconstruction.

## Methods

### Animal care and ethics statement

The Institutional Animal Care and Use Committee of the Abigail Wexner Research Institute at Nationwide Children’s Hospital (Columbus, OH) reviewed and approved the protocol (AR15‐00090). Animal care staff provided monitoring during all phases of the project. All animals received humane treatment following standards published by the Public Health Service, National Institutes of Health (Bethesda, MD) in the Care and Use of Laboratory Animals (2011) and US Department of Agriculture (USDA) regulations outlined in the Animal Welfare Act.

### Syngeneic tracheal graft (STG) harvest

Six to eight-week-old female C57BL/6 J mice were euthanized with an overdose of 200 mg/kg ketamine, 20 mg/kg xylazine, 10 mg/kg ketoprofen via intraperitoneal injection. A midline incision was made and the trachea was exposed. A 5-ring tracheal segment was excised and placed in chilled phosphate-buffered saline (PBS).

### Partially decellularized tracheal graft (PDTG) fabrication

Tracheal segments were collected as outlined in under “Syngeneic tracheal graft (STG) harvest”. Partially decellularized tracheal grafts (PDTG) were fabricated based on previously published methods^11,45^. Briefly, grafts were subjected to a graded sodium dodecyl sulfate (SDS) treatment before immersion in 1% TritonX-100 for 30 min at room temperature and 0.9% NaCl solution overnight at 4 °C. Partially decellularized tracheal grafts (PDTG) were stored in PBS (−20 °C) until implantation.

### Orthotopic tracheal graft implantation

Segmental tracheal replacement with PDTG and STG was performed in 6–8 week old, female C57BL/6 J mice as previously described in refs. ^29,46,47^. The animals used for transcriptomic analysis were euthanized at 2 weeks and 1 month. The animals used for histological analysis were euthanized at 1 month, 3 months and 6 months following implantation. Four animals of each graft type per time point were selected for analysis from this group.

### Post-operative care

Animals were provided buprenorphine (0.03 mg/kg, SC) and placed in a recovery cage on a heating pad until ambulatory. The animals were given ibuprofen (30 mg/kg) for 48 h in drinking water. They were observed for signs of respiratory distress, poor grooming, or weight loss. Unresolved respiratory distress or weight loss > 20% were criteria for early euthanasia.

### Euthanasia and sample collection

At the endpoint, animals were euthanized with an overdose of 200 mg/kg ketamine, 20 mg/kg xylazine, and 10 mg/kg ketoprofen via intraperitoneal injection. Following euthanasia, grafts were harvested and fixed in 10% neutral buffered formalin for at least 48 h at room temperature, embedded in paraffin, and sectioned at a thickness of 5 µm.

### Cell dissociation

Tracheal segments and explanted grafts were treated with dispase (3 mg/mL, ThermoFisher), collagenase (2 mg/mL, Sigma Aldrich), in 0.05% trypsin solution at 37 °C for 1 h^31,39^. Red blood cells and DNA were removed by sequential treatment with RBC Lysis Buffer (eBiosciences) for 5 min and 0.25 mg/ml DNAase for 10 min. Debris was removed by centrifugation through a fetal bovine serum-buffer step gradient. Single cells were resuspended in 1% BSA in PBS for RNA sequencing.

### Single cell RNA sequencing

An average of 10,000 cells per sample were processed on the Chromium Next GEM Single Cell 3’ kit at the Genomics Shared Resource at the Ohio State University Comprehensive Cancer Center and sequenced with Novaseq at the Institute of Genomic Medicine at Nationwide Children’s Hospital. Single-cell reads were demultiplexed and aligned to the mouse genome reference (ENSEMBL, mm8) using Cell Ranger (v.5.0.1, 10x Genomics) to obtain the unique molecular identifiers (UMI) counts per gene per cell matrix. For all data, quality control and filtering were performed to remove cells with a low number of expressed genes (threshold *n* > = 200), doublets, and elevated expression of apoptotic transcripts (threshold mitochondrial genes < 15%). Only genes detected in at least 3 cells were included. The expression profiles of the cells from different experimental groups were integrated separately before being clustered using the Seurat package (version 4.0) in R. Briefly, the counts were normalized, scaled and highly variable genes identified. Principal components were calculated and the dimensionality of the cells determined using the Elbow Plot Heuristic. Clusters of similar cells were detected using the Louvain method and visualized with Uniform Manifold Approximation and Projection (UMAP). A Model-based Analysis of Single-cell Transcriptomics (MAST) framework was used to identify differentially expressed genes from each cluster and canonical markers of various tracheal cell types were used to annotate the clusters^15–17^. Myeloid and epithelial cells were clustered to better define heterogeneity before combining them into a UMAP for visualization of all annotated cells.

### Trajectory inference

Trajectory inference on native, STG and PDTG groups was performed using Monocle 3 (package version 4.0.3)^21^. Briefly, a set of ‘landmark’ cells was selected using the kmeans() clustering algorithm in R and a principal graph that defines the trajectories was learned within the landmark cells. The principal graph was used as a guide to contract a graph on all the cells where the pseudotime of each cell can then be computed back to assigned root node. Genes that vary in expression over a developmental trajectory was then identified using the graph test() function. Plots were generated within Seurat and Monocle3 or using the ggplot2 package.

### Histology

Specimens were sectioned longitudinally to visualize both the host and graft regions of the airway and assessed via hematoxylin and eosin (H&E) staining. Additional sections then underwent immunofluorescence with quantification of basal progenitor cells (Keratin 5 (K5+), Keratin 14 (K14+), Antigen Ki-67), differentiated epithelial cells (Club Cell Secretory Protein (CCSP), Acetylated Tubulin (ACT+)), and endothelial cells (CD-31). A counterstain of 4,6-diamidino- 2-phenylindole (DAPI, Invitrogen, CA, USA) was used.

### Keratin5 and Keratin14 immunofluorescence and quantification

Rabbit Anti-K5 (1:1000 dilution ratio, Biolegend) was the primary antibody used for K5 and Mouse Anti-K14 for K14 (1:250 dilution ratio, Invitrogen). Immunofluorescence for K5 was detected using Alexa Fluor 594 donkey anti-rabbit IgG (1:500 dilution ratio, Invitrogen) and Alexa Fluor 488 goat anti-mouse IgG (1:500 dilution ratio, Invitrogen). Localization of K14-positive activated basal epithelial cells in the graft and anastomotic regions were determined by qualitative analysis. Both K5 and K14-positive basal cells were measured as a function of cells per mm of the basement membrane of the graft using ImageJ software [Rasband, W.S., ImageJ, U.S. National Institutes of Health, Bethesda, Maryland, USA, https://imagej.nih.gov/ij/, 1997–2018]. Further evaluation of K5+ basal cells was conducted on the mid-graft of STG. The mid-graft of the STG is defined by the length of the middle cartilage along with its 2 adjacent intercartilagenous spaces.

### Ki67 immunofluorescence and quantification

The primary antibody used for Ki67 immunofluorescence staining was Rat/anti-Mouse Ki67 (1:200 dilution ratio, Invitrogen). Immunofluorescence for Ki67 was detected using Alexa Fluor 647 donkey anti-rat IgG (1:500 dilution ratio, Invitrogen). Cells were quantified as a percentage of K5+/Ki67+ over all K5+ cells using ImageJ software.

### Club cell secretory protein (CCSP) and Acetylated alpha-tubulin (ACT) immunofluorescence and quantification

Immunofluorescent staining for club cells was performed with goat anti-Club cell secretory protein (CCSP) (1:200 dilution, graciously provided by co-author SDR^48^); ACT was detected with Mouse anti-acetylated α-tubulin (1:8000 dilution, Invitrogen). Immunofluorescence for CCSP was detected using Alexa Fluor 488 donkey anti-goat IgG (1:500 dilution ratio, Invitrogen) and ACT was detected using Alexa Fluor 488 goat anti-mouse IgG (1:500 dilution ratio, Invitrogen). CCSP+ cells were counted as a function of cells per mm of the graft; ACT was quantified as % coverage of ACT+ cells on the basement membrane of the graft. Further evaluation of CCSP and ACT was conducted on the mid-graft of STG as defined above.

### CD31 immunofluorescence and quantification

CD31 immunofluorescence staining was performed using Rabbit anti-CD31 (1:200 dilution ratio, Abcam). Immunofluorescence for CD31 was detected using Alexa Fluor 488 Goat anti-rabbit IgG (1:500 dilution ratio, Invitrogen). CD31+ cells were quantified as cells per 100 µm x 100 µm (high powered field (HPF)).

### Mass spectrometry

Native trachea (*N* = 4), and PDTG pre-implantation (*N* = 4) was assessed using mass spectrometry. Briefly, samples were digested by adding 5% SDS solution in 50 mM Triethylammonium bicarbonate (TEAB) solution and lysed with probe and bioruptor sonicators. Protein concentration was measured using Qubit (Thermo Fisher Scientific). Twenty µg of protein from each sample was digested using S-Trap™ sample processing technology (Protifi, Farmington, NY, USA). Two μg of protein underwent liquid chromatography followed by tandem mass spectrometry (LC-MS/MS). Data were analyzed using MASCOT via Proteome Discoverer™ (Thermo Fisher Scientific, Waltham, MA, USA), summarized using Graft software (Proteome Software, Inc., Portland, OR, USA), and compared using Microsoft Excel. Gene ontology annotations were obtained with Graft software. Spectra count were used as a semiquantitative measure of protein abundance.

### MicroCT and image segmentation

MicroCT was performed on live mice as previously described in ref. ^11^. In vivo imaging was performed with the Trifoil eXplore Locus RS 80: animals were positioned prone in the microCT chamber under inhalational anesthesia (1–3% isoflurane in room air at 1–3 L/min). All scans had full resolution reconstruction, producing 45 µm sections for living animal scan. The reconstructed microCT images were segmented using ITK-SNAP 4.0 software (http://www.itksnap.org/pmwiki/pmwiki.php).

### Statistical analysis

Data normality was evaluated by the Shapiro-Wilk test (S-W). Data with normal distribution were analyzed with ordinary one-way ANOVA and Tukey’s multiple comparisons tests. Data without normal distribution were analyzed with Kruskal-Wallis and Mann-Whitney t-test. All analyses were performed with GraphPad Prism 8.0 (GraphPad Software Inc., CA. USA). Quantified immunofluorescence data were expressed as mean ± standard deviation (SD).

## Supplementary information

Supplementary data 1

## Data Availability

The data that support the results of this study, experimental protocols, and additional details regarding methods employed in this study will be made available through request with the corresponding author (TC). The raw scRNA-seq datasets of mouse tracheal tissue (fastq format) have been deposited to NCBI-SRA with the accession number PRJNA954770.

## Code availability

The underlying code for this study may be made available to qualified researchers on reasonable request from the corresponding author.
